# An EEG database for the cognitive assessment of motor imagery during walking with a lower-limb exoskeleton

**DOI:** 10.1038/s41597-023-02243-7

**Published:** 2023-06-02

**Authors:** Mario Ortiz, Luis de la Ossa, Javier Juan, Eduardo Iáñez, Diego Torricelli, Jesús Tornero, José M. Azorín

**Affiliations:** 1 Brain-Machine Interface System Lab, Miguel Hernández University of Elche, Elche, 03202 Spain; 2 Instituto de Investigación en Ingeniería de Elche-I3E, Miguel Hernández University of Elche, Elche, 03202 Spain; 3 Instituto Cajal, Spanish National Research Council (CSIC), Madrid, 28002 Spain; 4 Center for Clinical Neuroscience, Hospital los Madroños, Brunete (Madrid), Madrid, 28690 Spain; 5 Valencian Graduate School and Research Network of Artificial Intelligence - valgrAI, Valencia, Spain; 6 Present Address: Center for Clinical Neuroscience, Hospital los Madroños, Brunete (Madrid), Madrid, 28690 Spain

**Keywords:** Cognitive control, Attention

## Abstract

One important point in the development of a brain-machine Interface (BMI) commanding an exoskeleton is the assessment of the cognitive engagement of the subject during the motor imagery tasks conducted. However, there are not many databases that provide electroencephalography (EEG) data during the use of a lower-limb exoskeleton. The current paper presents a database designed with an experimental protocol aiming to assess not only motor imagery during the control of the device, but also the attention to gait on flat and inclined surfaces. The research was conducted as an EUROBENCH subproject in the facilities sited in Hospital Los Madroños, Brunete (Madrid). The data validation reaches accuracies over 70% in the assessment of motor imagery and attention to gait, which marks the present database as a valuable resource for researches interested on developing and testing new EEG-based BMIs.

## Background & Summary

The EUROBENCH project emerges with the idea of creating the first unified benchmarking ecosystem for robotic systems in Europe (https://eurobench2020.eu/). The project is mostly focused on wearable robots and humanoids and aims to integrate the state of the art technologies for the assessment of these robotic systems into one unified methodological and experimental framework. The methodological components include a set of standard experimental protocols, a tool for the automatic performance score assessment of the robotics, a register’s database of experiments carried out in the facilities and an online platform to use the data processing algorithms. All of them have been included in a software suite to be used worldwide by researchers, developers and end-users^1^. The experimental framework is composed of two testing facilities, one for exoskeleton and prostheses located in Madrid (Spain) and a second humanoid-oriented located in Genoa (Italy). These benchmarking tools have been offered to beta testers during a validation campaign, in which different subprojects were offered the opportunity to use the software and the facilities.

The DECODED subproject, as one of the subprojects funded by EUROBENCH, participated in the validation of the benchmarking framework through two different scenarios. The experimental settings employ a lower-limb exoskeleton on two surface conditions: flat and inclined terrain. The subproject expands the original scenarios adding a Brain-Machine Interface (BMI) to the setup and the electroencephalography (EEG) recordings. One of the objectives was to test our previous developed BMIs^2–5^ and improved them through the new recordings carried out in the EUROBENCH’s facility.

The use of lower-limb robotic exoskeletons could help individuals with motor limitations. They can provide assistance and improve bottom-up rehabilitation thanks to the assisted motion executed by the device. The usability and clinical relevance of these robotics systems could be further enhanced by BMIs due to the top-down approach as they can improve neuroplasticity mechanisms^6–8^ through the cognitive engagement of the patient. A BMI makes context-based decisions based on the decoding of brain activity through non-invasive electroencephalographic (EEG) recordings. Different approaches have been explored in the last decade to interact with robotics exoskeletons by using BMIs based on EEG^9,10^. One of the most suitable paradigms to control an exoskeleton is motor imagery (MI). Although gait is a passive mental action that does not usually require the actual cognitive engagement, a BMI based on MI allows a direct and voluntary operation of the devices beyond the diminished physical capabilities of the subject. The focus of the subject on the mental task of motion provides a more intuitive control of the device in comparison to external interfaces such as joystick and buttons^9^, the monitoring of the torque^11^ or other EEG techniques based on Steady State Visual evoked Potentials (SSVEP)^12^. In addition, the involvement of the patient during rehabilitation techniques has the potential to enhance the brain restoration^7^. Literature demonstrates that the mental task of imaging a movement produces similar brain patterns than the executed motion^13,14^. Summing up, a BMI based on MI would be more intuitive and help the patient to be focused on the therapy.

However, the accuracy of the decodification of the brain signals must be improved not only in clinic environments, but also at home or outdoors. One of the main problems is that the subject is susceptible to distractions. Therefore, cognitive engagement plays a key role for the successful decodification of MI. Databases that include subjects’ EEG signals while they are controlling exoskeletons are not common, so advances in the development of BMIs for controlling exoskeletons have been progressing slowly in the last years compared to other research fields.

This makes the EEG database associated with this article a valuable contribution to research. Indeed, one of the objectives of the DECODED subproject was to provide the EUROBENCH database with EEG recordings valid for the cognitive assessment of MI during the use of a lower-limb exoskeleton. This is reflected on the current research as two different performance indicators (PI) which will be presented in the technical validation section: motor imagery (MI) and attention to gait (Att). However, the contribution of the database is wider as it could help researchers not only to compare their own MI and Att algorithms, but also to develop and test artifact mitigation techniques based on electrooculography channels, such as^15^, or motion^16,17^. Database could also be used to study the transitory of walk marked by an external cue as an event related potential (ERP)^18^ or the influence of surface inclination in the brain patterns.

EUROBENCH looks for creating an unified database with an standard format in the scope of bipedal robotics. However, it does not consider a specific format for EEG signals, so an adaptation of the format from our former architecture developed in © MATLAB to the .csv format was needed as this paper will explain. Due to the specific protocols designed, more robust BMIs could be developed in the future. The paper shows a pioneer database that will help researchers worldwide to develop, test and compare their decoding algorithms based on the actual cognitive engagement of the subject. This will be done not only when the subject is wearing the a lower-limb exoskeleton (H3 Technaid, Spain) on a flat surface, but also during different angles on ascending and descending slopes. The designed protocols are similar to the one presented in a previous research which used the Rex (Rex Bionics, USA)^5^, but adapted to the H3 exoskeleton (Technaid S.L., Spain) and with an specific protocol for non-flat surfaces.

## Methods

This section will describe the equipment used for recording the data and the protocols used for both scenarios. An explanation of the data preprocessing techniques and the software designed for the registering will also be included.

### Equipment

**Fig. 1:**
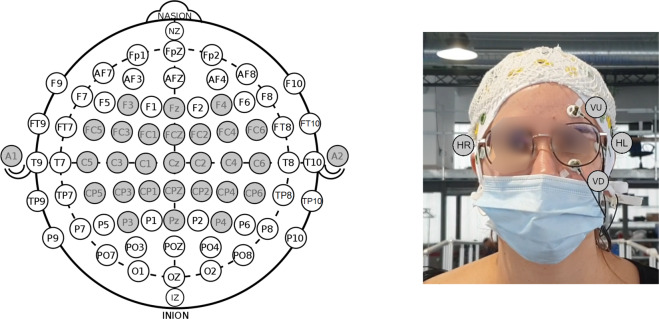
Distribution of the EEG electrodes based on International 10-10 system. 27 electrodes were used for EEG recording and 4 for EOG acquisition. Ground (A2) and reference (A1) electrodes were positioned on the ear lobes.
- The exoskeleton used was a H3 (Technaid, Madrid, Spain). It was configured for 100% assisted walking. For additional stability, all the subjects were helped by crutches. Any risk of fall caused by unbalance loss was prevented by a member of the technical staff positioned behind. Commands for the established protocol were send via bluetooth by the self-made software developed in © MATLAB.

As indicated before, two different protocols were developed to assess the cognitive engagement during the use of a lower-limb exoskeleton depending on the walking surface: flat terrain (Experience), inclined terrain (Slopes). Both protocols used the following systems:

- For the recording of the electroencephalographic signals (EEG), a bundle of 32 wet slim electrodes was used (Brain Products GmbH, Germany). Twenty seven of the electrodes were positioned with the help of suited sized caps (actiCAP, Brain Products GmbH, Germany) using the 10-10 system (F3, FZ, FC1, FCZ, C1, CZ, CP1, CPZ, FC5, FC3, C5, C3, CP5, CP3, P3, PZ, F4, FC2, FC4, FC6, C2, C4, CP2, CP4, C6, CP6, P4). Four of the electrodes (VU, VD, HR and HL) were placed in bipolar setup for electrooculography (EOG) acquisition, vertical electrodes around left eye. Ground and reference electrodes were located in the right (A2) and left (A1) ear lobes respectively. See Fig. 1 for further detail. The data were sampled at a 200 Hz pace. To avoid any possible interference that could affect the registered data, signals were sent wired to an actiCHamp amplifier (Brain Products GmbH, Germany). It was placed on a cart or inside a bag carried out by a member of the staff placed behind the subject depending on the scenario.

During the Experience experiments, additional physiological parameters were registered by the help of: Zephyr BioHarness 3.0 (Medtronic plc, USA), to register heart rate (HR), heart rate variability (HRV) and respiration rate (RR); and Shimmer 3 GSR (Shimmer Research Ltd, Ireland), to register the galvanic skin response (GSR) of the subject. All the signals were synchronized by the timestamp of the computer. These data are not included in the present paper as they are not used for EEG decoding of MI and attention, but will be incorporated into the EUROBENCH’s own database once operative (check https://eurobench2020.eu/ for updates in this regard) for assessing other performance indicators such as fatigue or focus attention.

In addition, the Slopes protocol used a custom platform that allows to change the slope’s angle by one degree steps. This scenario also employs seven Tech-IMUs CV4 (Technaid S.L., Spain) and 16 Trigno Avanti EMG recording sensors (Delsys Inc., USA), distributed throughout the legs. As in the Experience experiments, these recordings are not provided in the database of this article as they are not related to EEG decoding, but they will also be public on the EUROBENCH platform when finished.

**Fig. 2:**
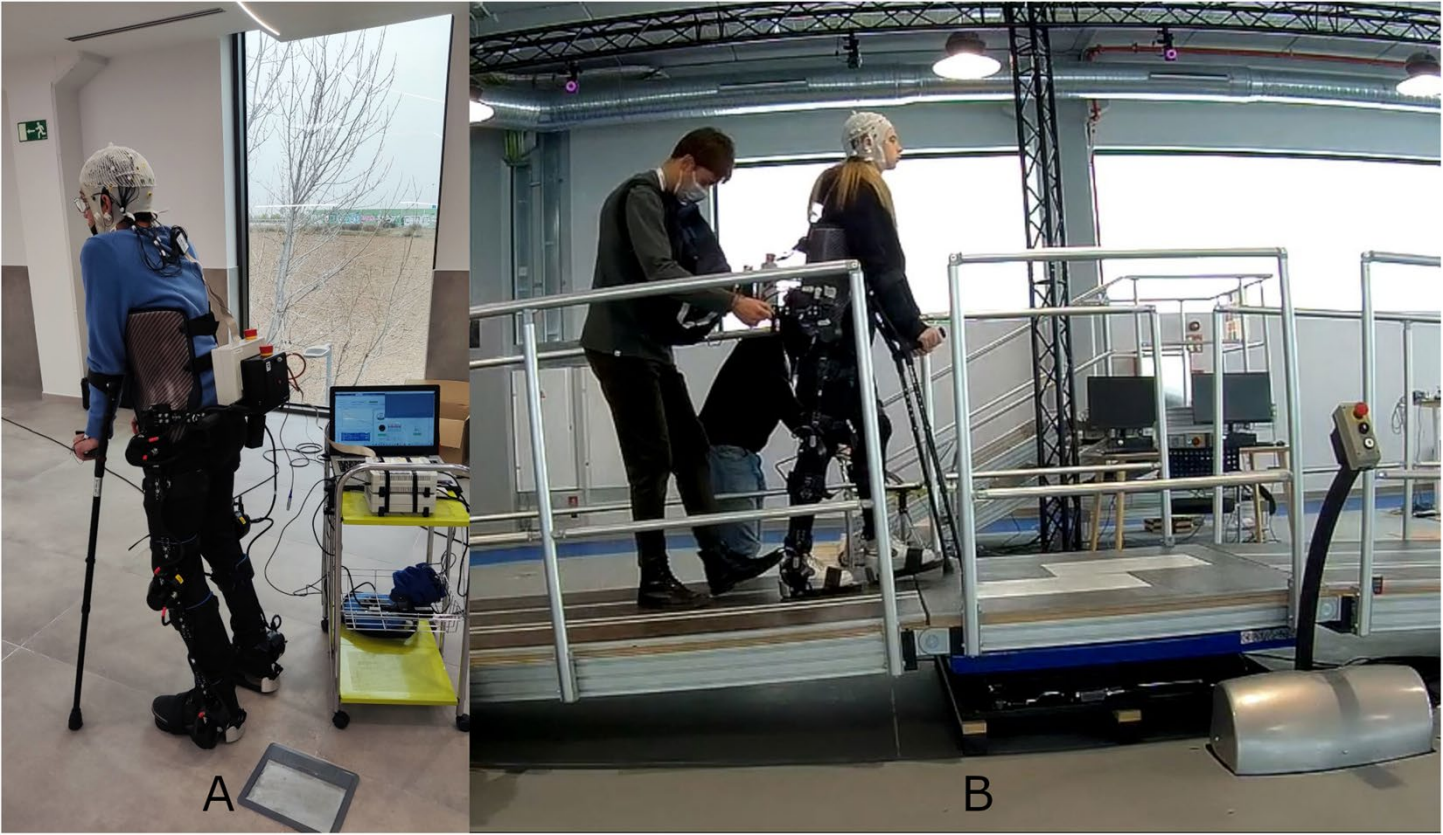
Equipment setup. (**a**) Experience scenario. The amplifier and battery of the EEG acquisition system was placed on a cart pushed by a member of the staff. Another member provided also additional support to prevent any risk of balance loss (not present in the image for better visualization of the setup). The subject used crutches as they are required by the H3 exoskeleton. (**b**) Slopes scenario. The setup is similar to Experience, except that the cart was parallel to the platform. The amplifier and battery of the EEG acquisition system was carried out in a bag by the member of the staff that provided balance stability. Other sensors were used in the experiments such as galvanic skin response and respiratory and heart beat rate monitoring. As these sensors were acquired by a third party software and are not relevant for the EEG study they were not provided in the database.

Figure 2 shows the equipment used during both kind of experiments.

### Protocols

The protocols were designed to record the EEG signals of subjects during the use of an overground exoskeleton over two different scenarios regarding the surface conditions, flat ground or a ramp. In order to assess the mental engagement during the use of the exoskeleton three different mental tasks were collected: relax, MI and regressive count. During relax, the subject was instructed to leave their mind blanked out, while during MI periods, the subject was focused on the kinesthetic imagery of the lower-limbs. In order to evaluate the attention to gait, the protocols were designed to collect the mental activity during gait while the subject was performing a high demanding mental task that assures them to be abstracted from the conscious walking in comparison to their mental activity during the MI focused gait. Previous investigations have tried other distracting activities such as video watching^19^ or varied mathematical operations^20^. However, in this research we opted for the regressive count indicated in our previous research^5^ as it does not require a screen which would be difficult to employ with the exoskeleton setup. Moving parts were recorded with the H3 exoskeleton at 100% assistance and the same speed level. However, due to the way the H3 exoskeleton manages speed, it can have minor variations due to the height of the subject. It is important to remark that the protocols were not designed to assess traditional MI, as the mental task of imaging motor action without any real movement would require full static registers, which is something we have already explored in previous investigations^3^. Therefore, MI class in this investigation should be considered as a high focused gait mental task during the use of the exoskeleton instead of the traditional definition of it.

#### Experience protocol (Exoskeleton-assisted overground walking)

Recordings were divided in three groups of trials:

- Four minutes trial while the subject is sat without wearing the exoskeleton.
- Four minutes trial while the subject remains standing up and wears the exoskeleton.
- Sixteen trials while the subject wears the exoskeleton and performs different mental tasks. The trials have static and movement periods. Original protocol consisted of 16 minutes of continuous walking on a treadmill, but as this original protocol did not allowed the required static periods and mental tasks, it was changed.

**Fig. 3:**
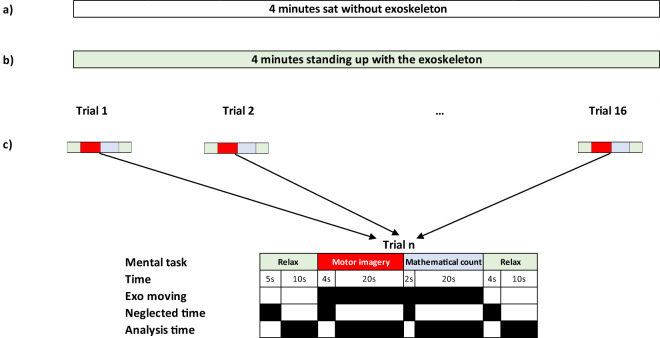
Experience protocol. It included three different kinds of trials. (**a**) Four minutes sat without exoskeleton; (**b**) Four minutes standing up with the exoskeleton; (**c**) 16 trials of different mental and physical activities.

The first two groups are provided as baseline trials (trials of four minutes), but are not used in the PIs calculation of our architecture. The third group, which consists of the remaining sixteen trials, was used for the PIs assessment and structured as follow, see also Fig. 3 for further information:

- 15 seconds of relax task, with the exoskeleton static and the user trying to stay as relaxed as possible, leaving their mind blank out. Five initial seconds were considered to allow the converging of posterior processing techniques. This meant that the effective time for analysis was 10 seconds.
- 24 seconds of MI task, with the exoskeleton moving and the subject focused on the kinesthetic imagination of the limbs movement. First two seconds covered the initial walking transition and two additional seconds were considered for neglecting any instructional evoked potential. This provided 20 seconds of effective signal for analysis. Instructions were given by voice command “Imagine”.
- 22 seconds of a regressive mental count task, with the exoskeleton moving. The user had to perform a cumulative subtraction of a big number (e.g. 900) minus a smaller number (typically between 5 and 10, but it was adjusted for every subject). Instructions were also given by voice command. The first two seconds considered the instructional evoked potential, leaving 20 seconds of time for analysis.
- 14 seconds of another relax task, with the exoskeleton stopped. First 4 seconds included the evoked potential and the time for the exoskeleton to stop, which left another 10 seconds of effective time of analysis.

#### Slopes protocol (Exoskeleton-assisted slope walking)

**Fig. 4:**
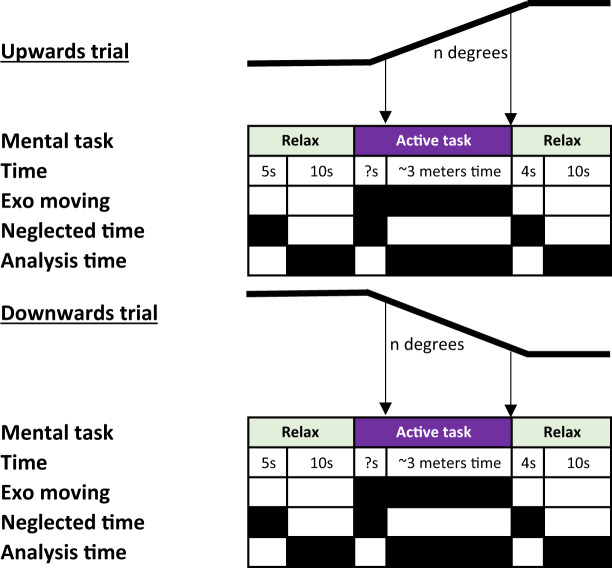
Slopes protocol. The image shows a couple of ramp up/down trials. Each couple, as the one showed, was repeated for each active mental task (MI or regressive count) and four angles from 1° to 4°. This way, the total number of trials were 16. To assure that the active analysis time was when the subject was on the ramp, task labelling cue was assigned based on visual inspection by a member of the technical staff. The arrows indicate the cue after starting the ramp and before finishing it. Time of active tasks were variable, because of that and the exoskeleton speed dependency on the weight and height of the subjects.

Due to the limited length of the ramp (5 m) some modifications were needed. First, as the ramp up/down periods of time can vary and are dependent on the speed of the exoskeleton, which is influenced by the subject’s height, the motion mental task (MI or regressive mental count) was carried out for each couple of ramp up/down trials. The slope began at 1 degree and it was increased one by one till 4 degrees after four consecutive trials (a couple of ramp up/down trials). This means that 16 trials were conducted in total (8 up/8 down). As the time to cover the ramps depends on the height and weight of the subject, event’s times for the active class (MI or regressive count) can be slightly different for each subject. The change to the active class was marked by visual inspection of the technical staff, after the exoskeleton stepped on the ramp/down and before finishing it. Figure 4 shows an scheme of the protocol.

### Subjects

Fourteen able-bodied subjects participated in the study (mean ± age, 28.7 ± 4.8). They did not report any known disease and had no movement impairment. All participants were right-footed, with 5 of them being female and 9 male. They were informed about the experiments and signed an informed consent form in accordance with the Declaration of Helsinki. All procedures were approved by the Ethics and Integrity in Research Committee of Miguel Hernández University of Elche (Spain) (Reference DIS.JAP.05.20) and the Ethics Committee of CSIC (Madrid, Spain) (Internal reference 091/2021). Consent for video and image recording was also given.

The experiments were carried out during different weeks and not all the subjects participated in both scenarios and fulfilled both sessions. Table 1 shows the information of the participants and the sessions they were engaged.

**Table 1:** Distribution of the sessions carried out by each of the subjects for both scenarios.

Subject	CSV code	Mat Code	Experience		Mat Code	Slopes	
			sep-21	mar-22		dec-21	apr-22
			oct-21				
			W1	W2		W1	W2
**S1**	Subject_01	M05	yes	yes			
**S2**	Subject_02	M06	yes				
**S3**	Subject_03	M07	yes				
**S4**	Subject_04	M08	yes	yes	M17		yes
**S5**	Subject_05	M09	yes		M13	yes	
**S6**	Subject_06	M10	yes		M15	yes	
**S7**	Subject_07	M11	yes	yes			
**S8**	Subject_08						
**S9**	Subject_09				M14	yes	yes
**S10**	Subject_10				M16	yes	yes
**S11**	Subject_11				M18	yes	
**S12**	Subject_12				M19		yes
**S13**	Subject_13	M20		yes			
**S14**	Subject_14	M21		yes			

The experimental sessions were carried out in two different weeks per protocol in the Eurobench complex (W1 and W2). Please check the *.mat files name to see the actual date of each recording. Although S8 signed the informed consent and started the experiments for a W2 slope test, technical problems with the acquisition equipment did not allow to finish the experiment. Therefore, data were not uploaded to the database for S8 subject.

### Data pre-processing

Provided data were pre-processed only by the hardware filters assigned in the pycorder software working with the EEG acquisition equipment. The assigned filters were a 0.1 Hz high-pass filter to avoid the DC component and a 50 Hz notch filter to mitigate the network component contribution. Data were sent to the custom EEG acquisition architecture in © MATLAB by an API that was slightly modified from the original one provided by Brain Products. No further pre-processing was applied to the data to keep them as much raw as possible and allow the users of the database to apply their own artifact removals techniques. Electrodes and wires movement were limited thanks to the use of a medical mesh as it is shown in Fig. 1. This was proved as useful in our previous investigations^4,5^.

Nevertheless, some actions were considered in order to allow the use of artifact removal techniques such as the two trials of four minutes baseline for the use of Artifact Subspace Reconstruction (ASR)^16^ or Independent Component Analysis (ICA)^21^. Besides, four EOG channels were registered to allow eye artifact removal techniques such as the one presented by Kilicarslan *et al*.^15^. In addition to the time needed to connect and synchronize equipment (label 0 in the .mat files), trials start with an initial five seconds length period to allow the convergence of state variable filters, such as the band-pass filters used in our processing, or other preprocessing techniques that require converge time, such as EOG artifact mitigation^15^.

## Data Records

Data are provided as two different datasets in figshare platform^22^. Original data include the .mat files that were registered using the custom software developed to work with © MATLAB. They include multiple variables not relevant for the dataset, as the software acquisition architecture was designed to work with other devices and algorithms. Nevertheless, in order to facilitate the use of the files by third party researchers, they were simplified and exported to .csv format limiting the information to only the relevant variables and making them not dependant on © MATLAB software.

### Repository

Datasets are available on figshare^22^.

### Data format

Each of the experimental trials was originally recorded as an individual .mat file and converted to .csv format.

**Fig. 5:**
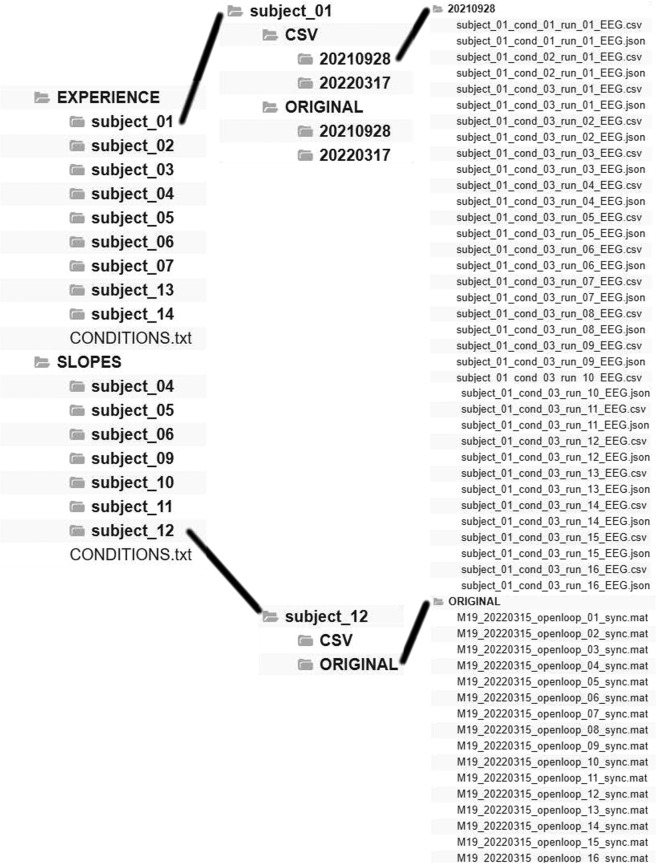
Distribution of files in folders.

The folder structure is organized in different levels. This can be seen in Fig. 5. First level indicates the scenario used for the experimental sessions: Experience or Slopes. Next folder represents the code of the subject used (subject 01 to subject 14). Then, files are organized by format in CSV and ORIGINAL folders. Inside of these folders the files can be directly found if only one session was carried out per scenario. In case, a subject performed more than one session, see Table 1, a folder named as the date of the experiment (year,month,day) contains the files.

#### Original data

Each of the original registered trials were saved as a © MATLAB struct variable named session in a .mat file^22^. The register name follows the format **XXX_YYYYMMDD_openloop_NT_sync.mat** being: **XXX:** Subject code for .mat files, see Table 1; **YYYYMMDD:** date in Year-month-day format; **openloop:** Exoskeleton mode of control; **NT:** number of the trial registered and **sync:** a flag for the synchronization of other data acquisition items, so it appears only if other external devices were read such as Galvanic Skin Response, Inertial Motion Units or heart beat rate. As they are not provided in the database, this last mark is not relevant for the provided files.

Talking about the session struct, some of the fields are not used in this research. However, as they can be used in other investigations of the group, they are still present in the file structure. Depending on the file, not all of them are always present, e.g. if no exo was used “ EXO” subfields are not present. The most important struct fields are:

- **data_EEG** includes the data of the 27 EEG channels.
- **trigger_EEG** includes any possible trigger captured by the EEG amplifier in case it is used.
- **data_EXO** contains the reading of the exo status as it is readed from H3 exoskeleton in a code number.
- **trigger_EXO** contains any trigger sent by the H3 exoskeleton.
- **task_EEG** indicates the label names of the different events in the file by a code number.
- **task_index_EEG** marks the sample where a task event starts (positive) and ends (negative) based on the task order.
- **task_order_EEG** contains the samples were a task event happens by order.
- **event_EEG** contains any manual events marked by the technician in control of the acquisition tool.
- **data_preprocessed_EEG** contains the data EEG data as the first 31 rows and the data after any possible software preprocessing steps carried out in blocks of 31 rows. As in this research, no further preprocessing was applied, the variable contains exactly the same data than data EEG variable.
- **data_preprocessed_EXO** modifies the data EXO data in case any change is done to the status. As it was not the case, only zeros are saved.
- **results** contains result metrics when the architecture is used for data analysis. As only acquisition was performed, the variable is an empty matrix.
- **conf** consists of a struct that provides lots of information depending the way the software is used. As it was just used for acquisition, most of the subfields are empty or do not have any significance from the database point of view. Relevant information can be found mostly inside the acquisition subfield, which is also a struct. The more remarkable fields inside this substructure are:
  - acquisition.user_code: see Table 1.
  - acquisition.device.device_EEG: which provides important information in different fields about the hardware used for EEG and EOG acquisition, channel distribution and sampling frequency.
  - acquisition.task: it is another struct that includes among others the sequence of the tasks (coded by a number), the time in seconds and the task name in a list. For instance, task number 404 which lasts 20 s can be found in acquisition.task.task list as MotorImagery in Experience recordings.

#### CSV data

To simplify the use of the database by third party researchers, the original .mat files were converted to .csv format. The .csv files are named by the subject number as subject XX followed by the condition and run order, i.e. the number of the trial. Notice that in the files, trials are named as runs. Inside each scenario there is a CONDITIONS.txt file that clarifies these. In the case of Experience scenario they are:

- cond_01_run_01: 4 minutes of EEG register with the subject sitting without the exoskeleton. See a) in Fig. 3.
- cond_02_run_01: 4 minutes of EEG register with the subject standing wearing the exoskeleton. See b) in Fig. 3.
- cond_03_run_01:16: 16 trials of EEG register with the subject standing and walking wearing the exoskeleton, performing mental tasks. See c) in Fig. 3.

In the case of the Slopes scenario the condition number is associated with the inclination angle and the mental task and the run with the ramp up/down action. Although the information is indicated in CONDITIONS.txt file, it can be easily consulted in Table 2.

**Table 2:** Explanation of the CONDITIONS.txt for the Slopes scenario.

Inclination	cond	Mental Task	run	Action
1°	01	MI	01	Ascending
			02	Descending
	02	Regressive Count	01	Ascending
			02	Descending
2°	03	MI	01	Ascending
			02	Descending
	04	Regressive Count	01	Ascending
			02	Descending
3°	05	MI	01	Ascending
			02	Descending
	06	Regressive Count	01	Ascending
			02	Descending
4°	07	MI	01	Ascending
			02	Descending
	08	Regressive Count	01	Ascending
			02	Descending

Inclination degree depends on the condition number. Being odd conditions for MI and even for regressive count. The up/down walking was marked by the run number, i.e trial number.

Each of the individual trials is defined by two files, the proper .csv and a .json. The .csv file contains a matrix where each row is a sample. The columns are in order: the time in seconds, the 27 EEG electrodes in *μV*, the 4 EOG electrodes in *μV* and the label of the task. The .json includes the relevant information of each file, summing up the needed fields that are in the session.conf of the original recordings.

**Fig. 6:**
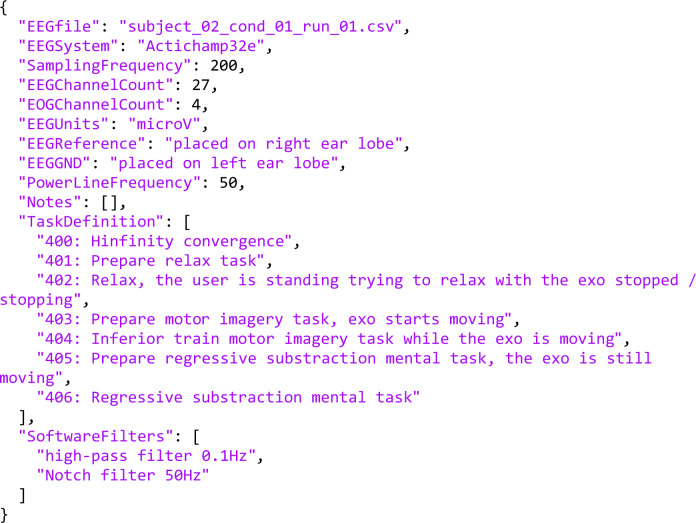
Example of the information provided in the.json files for a trial of Experience scenario.

The .json structure is the same for both scenarios. The fields are self-explanatory, including: the file name, the EEG acquisition system, the sampling frequency, the number of channels used for EEG and EOG acquisition, the units of the voltage acquired and the placing of the reference and ground. It also defines the labels associated with the different tasks carried out during the experiment and the pre-processing filters applied. An example of the .json structure can be seen in Fig. 6.

## Technical Validation

Two different paradigms are used to validate the database: MI and Attention to gait. For both paradigms two Performance Indicators (PIs) are calculated: MI index and Attention index. Both indices are tested for each of the individual files using the rest of them for training. Results are provided as the averaged accuracy of the leave-one-out cross validation for each subject. In addition, individual metrics and graphic representations per trial can be obtained through the use of the provided software for the calculation of the PIs. All the signals were processed as epochs of 1.5 seconds and shifted at a 0.5 seconds ratio. Notice that, as the methodology employed for technical validation of the data does not use low frequency bands, no ASR or EOG artifact removal techniques were applied.

### MI index

The aim of the algorithm is to create an index defining the MI level during the different mental tasks. The MI algorithm confronts two of the mental tasks conducted by the subjects during the experimental data collection: Relax and MI. The periods of time valid for the analysis are marked in Fig. 3 neglecting any transition events between tasks and their possible evoked potentials. Data are evaluated just for the 16 trials (trials c). The two starting trials of 4 minutes sat and standing up are provided just as a baseline useful for other data analysis, but they do not provide relevant information for the presented MI algorithm.

**Fig. 7:**
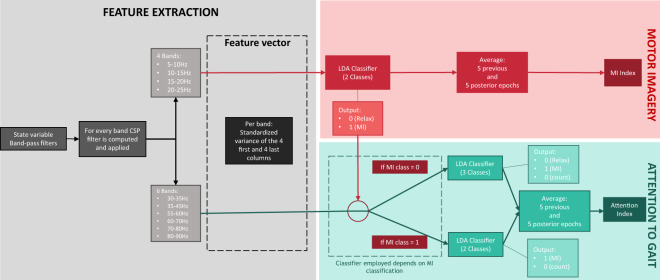
Details of the algorithms employed for the technical validation of the data and the calculation of MI and Att PIs.

The implemented algorithm is based on the previous research presented in^3^. A detailed flow diagram can be seen on the top right part of Fig. 7. Signals were band-pass filtered on four bands to define alpha and beta rhythms. For each band a common spatial filter (CSP)^23^ was computed, taking as extracted features, once standardized, the variance of the first and last four components.

After the feature extraction, a linear discriminant analysis (LDA) classifier^24^ was trained with the features of the two classes using the 15 data files, leaving one of the files for testing. In the case of the Slopes scenario, seven files were used for training the model leaving one for testing as eight were the files with MI conducted task, see Table 2. Each of the epochs of the testing vector was classified by a 0 (Relax) or a 1 (MI). This binary classification was averaged for the 5 previous and posterior shifted epochs in order to create the MI index. As it is computed epoch by epoch, it can be represented as a continuous vector with a resolution of one epoch. However, as it contains information of the 5 previous and posterior epochs, i.e. it contains information between [−3, +3] seconds, there is a natural delay in the MI index in the transition tasks, which are not used in the metric computation.

One of the main changes done to the original algorithm presented in^3^ are in the way the epochs are averaged. In addition, there is not a state-machine dual control classifier, as no real-time control decisions are needed in the current research, being the processing carried out in pseudo-online mode.

**Fig. 8:**
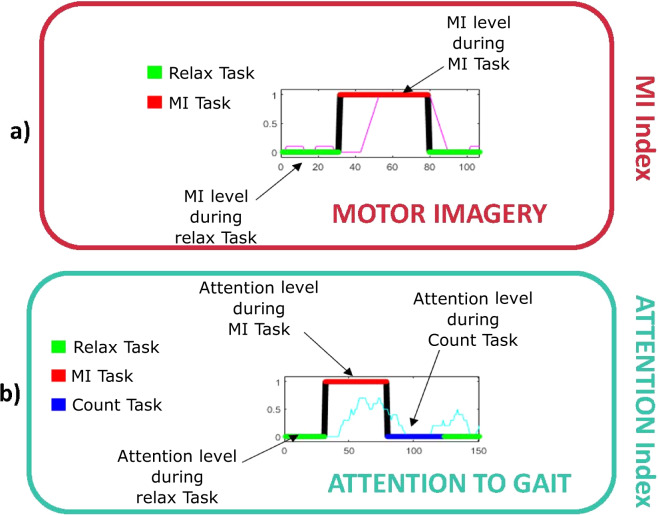
Example of the PI computed with the files. The images show two PI indices for a Experience trial. a) MI index for S1 and trial 6 (magenta). Notice that the signal in magenta is not represented for the regressive count period as it is not relevant for this PI; b) Attention index for S1 and trial 10 (cyan). As the PI is relevant not only for regressive count task, but also for MI and relax periods, the representation is shown for the whole trial. Although both PIs are shown for transition events of neglected time (see Figs. 3, 4), only the effective analysis time periods are considered for metric evaluation. X axis represents the epoch number which is shifted at a 0.5 seconds pace.

An example of MI index output can be seen in Fig. 8a). Notice that regressive count is not represented in the figure as the MI index uses a dual class classifier and regressive count task output is relevant only for the Attention PI. Slopes trials have only one active mental task per trial so the MI index output would be similar to the one shown for the 8 MI trials.

### Attention to gait index

The attention to gait (Att) has been studied in previous investigations^5,19,20^, demonstrating that differences on brain activity can be detected on gamma band during motion depending on the attention focus that the subject shows to the gait (high attention) or a distracting activity (low attention). Attention index has shown its usefulness as a correcting tool to limit the number of false positives in a BMI^5^ and its viability to work in a closed-loop control with a state machine control^3^. The algorithm used to assess the Att has been modified from previous investigations and uses CSP^23^ in a similar way to the MI index to extract the standardized variance of the first and last four columns of six sub-bands of gamma band, see Fig. 7. Besides the use of gamma band, the other difference with the MI algorithm is the use of a cascade classifier. This means that the CSP features are used to create or test two different classifiers depending on the MI paradigm output: two classes and two outputs (Count (0) vs. MI (1)), and three classes and two outputs (Relax + Count (0) vs. MI (1)), see Fig. 7 down. This conditional classification is first introduced in the current investigation along with the CSP gamma features improving the original results of the attention index.

Similar to the MI index, Att index is computed averaging the previous and posterior 5 epochs. An example of the Attention index output can be seen in Fig. 8b). Ideally, Attention to gait index should be low during the relax and regressive count periods and high during the MI ones. Equally to the MI index output, there is a inherent delay in the index due to the averaging of the previous epochs and it is influenced by transition tasks as it is computed as a continuous time vector during each trial.

### Metrics computation

**Fig. 9:**
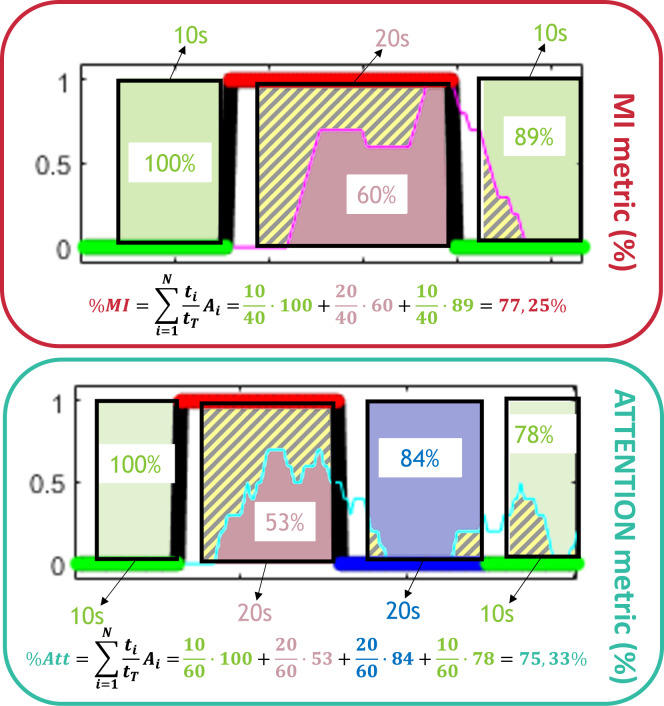
Example of the calculation of the PI metrics. The metric is assessed for the considered valid analysis time. These times are fixed for Experience trials and variable for the Slopes ones.

As both PIs are calculated as a continuous vector for each trial, a proper metric for the assessment of the results must be defined. Both PIs should be as high as possible during MI periods and low during the rest of tasks. Accuracy metrics are defined as the ratio of effective areas below or upon the PI during the valid periods of analysis, i.e. neglecting transition times:

1 $$ \% PI=\mathop{\sum }\limits_{i=1}^{N}\frac{{t}_{i}}{{t}_{T}}\cdot {A}_{i}$$

being *N* the number of periods, *t*_*i*_ the time of each period, *t*_*T*_ the total time analyzed and *A*_*i*_ the correct area ratio. Figure 9 shows an example of both PI metrics computation. The effective areas appear for each task color coded, leaving as striped areas wrong zones. Notice that as the PI indices have a certain delay, it is not possible to achieve 100% accuracies even for a perfect classification of all the epochs.

### Results

The metrics are computed for each tested file and given as the average and standard deviation of the leave-one-out cross validation of the trials for both scenarios in Table 3. For each tested file, the model is created with the information of the classes of the n-1 resting files. The data used for training the model corresponds to the windowed effective areas in Fig. 9. They are color coded for each of the mental tasks and associated with the data by their task name which are present in the *.json and *.csv files: green (relax task 402 in files), red (MI tasks 404/752/758 in files for experience, slopes up and slopes down respectively) and blue (tasks 406/756/758 in files for experience, slopes up and slopes down respectively). The indices are calculated as a continuous vector data, but assessed for the windowed effective areas as indicated in the metrics computation section. If individual accuracies want to be consulted, please check the results .mat file created by the technical validation software and the figures by trial that are generated.

**Table 3:** Accuracy results for the two experimental scenarios tested and both paradigms.

Subject	CSV code	Mat Code	Experience				Mat Code	Slopes			
			sep-21, oct-21		mar-22			dec-21		apr-22	
			W1		W2			W1		W2	
			MI	Att	MI	Att		MI	Att	MI	Att
S1	Subject_01	M05	79,2 ± 6,1%	76,9 ± 7,4%							
S2	Subject_02	M06	71,6 ± 5,6%	68,8% ± 5,7	78,3 ± 5,9%	73,8 ± 12,6%					
S3	Subject_03	M07	87,3 ± 4,9%	82,8 ± 4,6%							
S4	Subject_04	M08	80,0 ± 5,7%	73,1 ± 7,3%	76,5 ± 7,6%	76,2 ± 6,5%	M17			80,5 ± 7,3%	80,0 ± 11,0%
S5	Subject_05	M09	84,4 ± 4,5%	76,6 ± 5,3%			M13	81,4 ± 5,5%	83,3 ± 8,4%		
S6	Subject_06	M10	80,3 ± 5,4%	79,4 ± 10,6%			M15	84,2 ± 6,3%	79,1 ± 9,9%		
S7	Subject_07	M11	80,2 ± 6,2%	71,0 ± 6,1%	76,4 ± 5,0%	71,7 ± 6,2%					
S8	Subject_08										
S9	Subject_09						M14	80,1 ± 6,2%	80,6 ± 5,6%	82,2 ± 6,0%	82,0 ± 9,8%
S10	Subject_10						M16	75,0 ± 8,8%	79,6 ± 6,5%	72,1 ± 6,7%	73,9 ± 6,4%
S11	Subject_11						M18	71,3 ± 6,9%	75,6 ± 7,7%		
S12	Subject_12						M19			73,6 ± 8,7%	74,1 ± 10,0%
S13	Subject_13	M20			86,4 ± 4,8%	71,8 ± 6,4%					
S14	Subject_14	M21			76,8 ± 8,6%	72,7 ± 9,2%					
		Avg.	80,4 ± 4,9%	75,5 ± 4,9%	78,9 ± 4,3%	73,2 ± 1,9%		78,4 ± 5,2%	79,6 ± 2,8%	77,1 ± 5,0%	77,5 ± 4,1%

The results for the leave-one-out cross validation are calculated by subject and week of experiments.

**Fig. 10:**
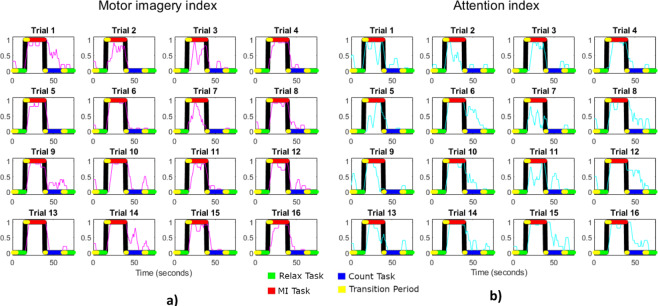
Computation of the indices of Experience protocol for subject S3 during the first week session. (**a**) MI index (magenta) for the sixteen trials registered, (**b**) Att index (cyan) for the sixteen trials registered. Indices range from 0 to 1. Time periods are color coded depending on the mental task executed and placed on 0 or 1 depending or the ideal value to obtain. Indices are computed for the whole trial including transition times (yellow). Model is trained with the information of the classes of the rest of the trials without using the transition periods 7.

As it can be seen, both paradigms show systematically accuracies over the 70%, which provides an indicator of the data validation. As an example to see the indices obtained per each individual trial Fig. 10 can be consulted.

The performance on flat surfaces (Experience) shows better results for the MI paradigm, showing a lower accuracy for all the subjects by almost a 5% in average for the Att index. In the case of the inclined surface (Slopes), both paradigms perform similarly without a significant difference. Comparing both scenarios, the Slopes scenario shows slightly lower results. However, this could be related to the high subject dependency that is present in the results and reported on previous investigations^2,5^. Indeed, the results are mixed when the comparison is done for subjects that participated in both scenarios. In the case of S4 the MI performance of slopes is similar for W1 Experience and higher than W2 Experience, while Attention paradigm performs better than the two Experience weeks. However, the behaviour of S5 is contrary to S6. While the MI performance of Slopes is better for S6, it is lower for S5, being the opposite for the Attention paradigm. All of this indicates that the minor differences in performance are just due to the normal EEG variations in performance expected when a BMI is analyzed.

There are not many investigations in the literature that assess the cognitive engagement or maintained MI during the use of an exoskeleton. Thus, the focus will be on previous iterations of the algorithms presented. The former research^5^ follows a very similar protocol to the one in this paper, but using a Rex exoskeleton (Rex Bionics, New Zealand) and Stockwell transform^25^ for MI paradigm and power spectral density by maximum entropy method^?^ for the attention paradigm. Results should be compared to the ones in the pseudo-online analysis for the MI + att pseudo-online analysis in Table 1^5^, as it is the one similar to the one conducted in this research. The average value for the indices are shown in Table 2 of the paper^5^ with a %MI of 71,2 ± 13,3% and %Att of 71,1 ± 10,3% (see Table 2) for the five sessions of the four subjects. These value are around 10 points lower and with a lower dispersion than the ones registered in the current research that achieves a %MI of 80,4 ± 4,9% (for week 1 sessions), 78,9 ± 4,3% (for week 2 sessions) and an %Att of 75,5 ± 4,9% (for week 1 sessions) and 73,2 ± 1,9% (for week 2 sessions). Slopes protocol shows also higher indices in average with lower dispersion, with a %MI of 78,4 ± 5,2% (for week 1 sessions), 77,1 ± 5,0% (for week 2 sessions) and an %Att of 79,6 ± 2,8% (for week 1 sessions) and 77,5 ± 4,1% (for week 2 sessions).

The research by Ferrero *et al*.^3^ introduces CSP for the computing of the MI index, keeping the maximum entropy method for attention assessment. It also uses the same H3 exoskeleton of this research. However, there is a significant difference in the used protocol, as it is designed to create a state machine for the closed-loop control of the exoskeleton in real time. This means that the control is designed to use the combination of two models depending on the exo status: static and motion. The most similar comparison to the research of the current paper will be with the results of the leave-one-out cross validation of the individual models during the training trials. As we are focusing on the cognitive engagement of the gait, the comparison will be done with the average of the sessions of the gait model in Tables 1, 2 of the paper^3^. They achieve a value for the average of the sessions performed of %MI 67,41 ± 12,00% and %Att 67,55 ± 2,44% for the first subject and %MI 56,17 ± 4,91% and %Att 64,5 ± 3,98% for the second subject. The results are lower than the ones of the current research, but it could be related to the subject dependency (just two subjects) and the protocol differences, as it is much harder to perform the blanked out mind relax event during gait than in a static situation.

The database could help worldwide researches to develop new EEG-based algorithms to assess the cognitive engagement in motor tasks when wearing a lower-limb exoskeleton not only on flat surfaces, but also on slopes, providing an EEG collection of recordings under a defined protocol that covers a gap in the research community.

## Usage Notes

### Mat2CSV converter

Create a /Registers/CSV folder in the directory the file is run. Use lines 13:14 for Experience files and lines 17:18 for Slopes ones. File tested on © Matlab R2022a version for the uploaded software^26^.

### DECODED PIs calculator

Please follow the instructions annexed to the technical validation software. The software is run for each subject and week. Create a /readfiles folder to place the csv files and a /results folder to allow the software to write the results. © MATLAB is required for the PI computation (tested on R2022a version).

Results include: the PIs per epoch in .csv and .mat files, figures with the continuous PIs indices and different area metrics computations under results.m file. The defined PI metrics exposed for the MI and Attention paradigms can be obtained per trial in the results.m for lines 9 and 12 respectively. For obtaining the results presented in Table 3, just type:

MI = mean(results(9,:))

Att = mean(results(12,:))

Please remember that the Experience files used in the technical validation are related to the condition 3 (16 trials), so just select cond03 files with the technical validation software. In the case of the Slopes scenario, all the 16 files of each session must be chosen.

## Data Availability

The file converter from original .mat files to formatted .csv files is available on figshare^26^. The code for the computation of the PIs is available on https://github.com/bmislab/DECODED.
